# Construction of Rheumatoid Arthritis-Associated Interstitial Lung Disease diagnostic model and identification of biomarkers based on a multi-omics integration strategy of machine learning

**DOI:** 10.1016/j.clinsp.2026.100933

**Published:** 2026-04-17

**Authors:** Dandan Wu, Jianghua Chen, Heng Liang, Cong Chen, Mei Liang, Cuiting Liao, Xueke He, Jiansheng Zhai, Min Dai, Xiaorong Lu, Fanxin Zeng, Qinghua Zou

**Affiliations:** a Department of Rheumatology and Immunology, First Affiliated Hospital of Army Military Medical University, Chongqing, China; b Dazhou Vocational College of Chinese Medicine, Dazhou, China; c Biobank, Southwest Hospital, Army Medical University, Chongqing, China; d People’s Hospital of Tongliang, Chongqing, China; e Department of Rheumatology and Immunology, Fengdu General Hospital, Chongqing, China; f Department of Clinical Research Center, Dazhou Central Hospital, Dazhou, Sichuan, China

**Keywords:** Rheumatoid arthritis, Interstitial lung disease, Multi-omics, Machine learning, Diagnostic model, Biomarker

## Abstract

**Objective:**

This study aimed to develop and validate a machine learning model integrating multi-omics and radiomics data to improve diagnostic accuracy and identify potential biomarkers for Rheumatoid Arthritis-Associated Interstitial Lung Disease (RA-ILD).

**Methods:**

A total of 278 patients with RA were enrolled across two cohorts. Cohort 1 (63 RA-nonILD, 46 RA-ILD) provided clinical data, chest CT images, plasma, and PBMC samples for non-targeted metabolomics, transcriptomics, and 4D DIA proteomics. Cohort 1 was split in a 6:4 ratio into training and validation sets. Machine-learning algorithms (RF, LASSO, SVM) and a Transformer model were used to screen biomarkers. Diagnostic models were constructed using LASSO, RF, LightGBM, and CatBoost. A combined imaging-clinical logistic regression model was developed and externally validated in cohort 2 (102 RA-nonILD, 67 RA-ILD). Associations between key biomarkers, inflammation, lung function, and CT severity were examined, and pathways related to the radiomic feature Kurtosis were explored.

**Results:**

Nine radiomic features, five metabolites, two proteins, and eight genes were identified as key biomarkers. The metabolomics-based CatBoost model showed the best single-omics performance (AUC = 0.982). The multi-omics integration model outperformed all single-omics models. The imaging-clinical model demonstrated strong diagnostic accuracy in both internal (AUC = 0.963) and external validation (AUC = 0.913), and a nomogram was constructed for clinical risk assessment. Key biomarkers correlated with inflammatory indicators and lung-function decline, and high-Kurtosis-associated genes were enriched in pro-fibrotic pathways.

**Conclusion:**

Integrating multi-omics and radiomics with machine learning yields a robust diagnostic strategy for RA-ILD. The imaging-clinical nomogram provides a practical tool for risk assessment, and identified biomarkers reflect disease severity and progression.

## Highlights

• The authors developed a high-accuracy RA-ILD diagnostic model by integrating multi-omics, radiomics and machine learning approaches.
• The clinical-radiomics nomogram shows robust diagnostic performance in both internal and multicenter external validation.
• Key biomarkers were identified that link molecular changes to inflammatory activation and pulmonary function impairment.
• Multi-omics integration outperforms single-omics strategies for RA-ILD diagnosis and reflects disease severity progression.

## Introduction

Rheumatoid Arthritis (RA) is an autoimmune disease characterized by chronic joint inflammation. Its most severe extra-articular complication, Rheumatoid Arthritis-associated Interstitial Lung Disease (RA-ILD), poses a grave threat to patient prognosis and quality of life.^1^ Studies have shown that approximately 10% of RA patients are diagnosed with RA-ILD using High-Resolution Computed Tomography (HRCT). However, after diagnosis, their median survival is only about 2.6-years, and the mortality rate is significantly higher than that of RA patients without ILD.^2^ This grim reality highlights the urgent need for early and precise diagnosis of RA-ILD.

At present, there are multiple bottlenecks in the differential diagnosis of RA and RA-ILD. First, RA-ILD presents with highly heterogeneous clinical manifestations, ranging from asymptomatic to Progressive Fibrosing ILD (PF-ILD). Approximately 50% of patients progress to PF-ILD, which greatly increases the difficulty of its early identification.^3^ Second, the core of the diagnosis relies on subjective visual assessment of HRCT findings, which is highly dependent on the radiologist's experience. Significant discrepancies exist in the interpretation of subtle pulmonary lesions, such as early interstitial inflammation and mild fibrosis, among different physicians. Furthermore, conventional HRCT lacks the sensitivity required to detect early pathological changes, resulting in frequent missed or incorrect diagnoses.^4^ Third, current potential biomarkers, such as Krebs von den Lungen-6, exhibit insufficient sensitivity and specificity. This limits their usefulness in supporting early diagnosis and accurate pathological stratification.^5^

Advances in omics technologies and artificial intelligence have made integrated multi-omics analysis and radiomics promising new approaches for diagnosing complex diseases.^6^^,^^7^ Multi-omics approaches that combine genomics, transcriptomics, proteomics, and metabolomics have been widely used to identify diagnostic and prognostic biomarkers in cancer. These methods provide a more comprehensive understanding of disease mechanisms and outperform single-omics analyses in biomarker discovery and patient stratification.^8^^,^^9^ Radiomics, involving the extraction of quantitative imaging features, has also demonstrated strong performance in predicting bone metastasis in breast cancer and prognosis in hepatocellular carcinoma.^10^^,^^11^ However, current research on RA-ILD remains constrained, as the majority of studies concentrate on either single-omics data or single-modal information, such as exclusively analyzing transcriptomic features or solely depending on HRCT imaging. This approach lacks a systematic integration of multi-omics molecular characteristics and radiomics information.^12^^,^^13^ As a result, it fails to fully cover the entire pathological process of RA-ILD, from molecular abnormalities to tissue pathological changes. It also cannot fully leverage the synergistic effects of multi-modal data in enhancing diagnostic accuracy and exploring disease mechanisms.^14^

In this study, the authors integrated peripheral blood transcriptomics, proteomics, metabolomics data, HRCT imaging, and clinical indicators. The authors then constructed and compared various diagnostic models to achieve accurate differentiation between RA and RA-ILD, while also identifying potential diagnostic biomarkers associated with RA-ILD. The aim of this study was to overcome the limitations of traditional HRCT-based diagnosis, improve the accuracy and objectivity of differentiating RA from RA-ILD via multimodal data integration analysis, and thus provide new biomarkers for the early screening of RA-ILD.

## Materials and methods

### Research subjects and ethics

**Fig. 1:**
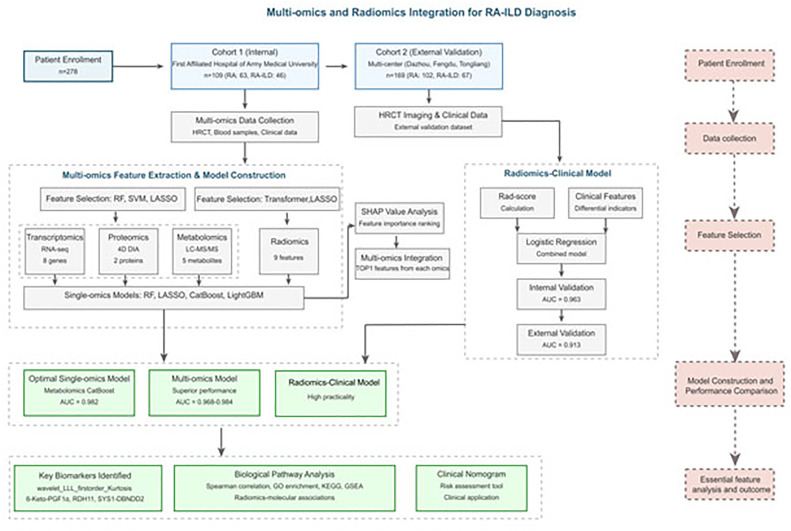
Flowchart of this study. AUC, Area Under the ROC Curve; CatBoost, Categorical Boosting; HRCT, High-Resolution Computed Tomography; LASSO, Least Absolute Shrinkage and Selection Operator; LightGBM, Light Gradient Boosting Machine; RA, Rheumatoid Arthritis; RA-ILD, Rheumatoid Arthritis-Associated Interstitial Lung Disease; RF, Random Forest; ROC, Receiver Operating Characteristic; SVM, Support Vector Machine.

This study was approved by the Ethics Committee of the First Affiliated Hospital of Army Medical University [(B) KY2025065]. The study was conducted in accordance with the Standards for Reporting of Diagnostic Accuracy Studies (STARD) guidelines to ensure the completeness and transparency of diagnostic research reporting. The authors retrospectively recruited 269 patients from four hospitals between January and June 2025. Cohort 1 (*n* = 109; 63 RA, 46 RA-ILD) from the First Affiliated Hospital of Army Medical University served as the internal dataset and was randomly allocated into training and validation sets (6:4 ratio). Cohort 2 (*n* = 160; 102 RA, 67 RA-ILD) was pooled from Dazhou Central Hospital, Fengdu County People's Hospital, and Tongliang District People's Hospital to serve as an independent external test set. Inclusion criteria required: 1) Age ≥ 18-years; 2) Compliance with the 2010 American College of Rheumatology (ACR)/European League Against Rheumatism (EULAR) classification criteria for RA^15^; 3) Complete, high-quality HRCT scans; 4) RA-ILD diagnosis confirmed by a multidisciplinary team (radiologists, rheumatologist, pulmonologist) based on characteristic HRCT findings; and 5) Complete clinical data. Exclusion criteria included concurrent autoimmune diseases, other lung pathologies (e.g., tuberculosis, malignancy), severe organ dysfunction, pregnancy/lactation, or severe psychiatric/cognitive disorders. The study design is illustrated in Fig. 1.

### Multi-omics data collection and preprocessing

#### Radiomics analysis

To standardize imaging data across centers, all HRCT images were resampled to a 1 × 1 × 1 mm^3^ voxel size and normalized to a standard lung window (level −500 HU, width 1500 HU). Regions of Interest (ROIs) covering interstitial lesions were semi-automatically segmented using 3D Slicer. Segmentation consistency was validated by two blinded senior radiologists, with a Dice similarity coefficient ≥ 0.95 considered consistent. Radiomic features were extracted using PyRadiomics and standardized via *z*-score normalization. A Vision Transformer (ViT) model was employed for dimensionality reduction to generate high-level feature representations. The Least Absolute Shrinkage and Selection Operator (LASSO) regression (10-fold cross-validation) was then used to select optimal features and calculate the Rad-score.

#### Transcriptomic analysis

Peripheral blood samples were collected from the participants, and total RNA was extracted and subjected to quality control. The samples were sent to Shanghai Meiji Biomedical Technology Co., Ltd. for library construction and sequencing. Paired-end sequencing was performed using the Illumina platform. After obtaining the raw data, adaptor trimming, quality filtering, and alignment to the human reference genome GRCh38. p13 were conducted. Transcript assembly and differential expression analyses were performed using StringTie and DESeq2 (screening criteria: |log₂FC|>1, *p* < 0.05).

#### Proteomics analysis

Protein extraction was performed using lysis buffer containing urea and SDS. After quantification using the BCA method, SDS-PAGE electrophoresis was performed. Peptide fragments digested with trypsin were desalted and analyzed using a Vanquish Neo mass spectrometer in the Data-Independent Acquisition (DIA) mode. Raw data were searched and quantified using Spectronaut software, with the protein False Discovery Rate (FDR) set at ≤0. 01.

#### Metabolomics analysis

Fasting serum samples were collected and pretreated with methanol precipitation, followed by LC-MS/MS analysis using the UHPLC-Q Exactive HF-X system. Mass spectrometry data were preprocessed using Progenesis QI software, and metabolites were identified and quantified using the HMDB and Meiji Biology Metabolite Databases.

### Feature selection and machine learning model construction

Candidate biomarkers were identified by intersecting features selected by three algorithms: LASSO, Support Vector Machine (SVM), and Random Forest (RF), following preliminary screening via Partial Least Squares Discriminant Analysis (PLS-DA). Using these key features, single-omics diagnostic models were constructed using LightGBM, CatBoost, LASSO, and RF algorithms. Model performance was evaluated in the internal validation set using AUC, accuracy, sensitivity, and specificity, and interpreted using SHapley Additive exPlanations (SHAP). Additionally, an integrated Multi-omics Model was built using the top-ranked features from each omics layer. For clinical application, the authors developed a logistic regression-based Clinical-Radiomics Model incorporating the Rad-score and significant clinical features.

### Statistical analysis and biological interpretation

Statistical analyses were performed using R (v4.5.0) and SPSS (v22.0). Continuous variables were compared using Student's *t*-test or Mann-Whitney *U test*, and categorical variables using Chi-Square tests. Spearman's rank correlation (adjusted for False Discovery Rate [FDR]) was performed to assess relationships between key multi-omics/radiomics features and: 1) Systemic inflammatory markers (e.g., CRP, ESR, NLR, SII); 2) Disease severity indices in RA-ILD (e.g., FVC%pred, DLCO%pred, Warrick score, CPI); and 3) Molecular data (differentially expressed genes/metabolites). Biological functions were explored via Gene Ontology (GO) enrichment for correlated genes and KEGG pathway analysis for metabolites. Gene Set Enrichment Analysis (GSEA) was conducted to identify pathways associated with high-risk radiomics phenotypes. Significance was set at *p* < 0.05 (two-tailed) or FDR <0.05.

## Results

### Baseline characteristics of study subjects

A total of 109 patients were included in Cohort 1 (Table 1), comprising 63 RA patients and 46 RA-ILD patients. There were no significant differences between the two groups in terms of age, gender, smoking history, or joint activity indicators (number of tender/swollen joints and DAS28-ESR score). The RA-ILD group had significantly higher pain VAS scores, Alkaline Phosphatase (ALP), urea, and creatinine levels than the RA group. Levels of Rheumatoid Factor (RF) and anti-CCP antibodies were also significantly elevated. No statistically significant differences were observed between the two groups in terms of hematological, liver and kidney function, or metabolic parameters.

**Table 1:** Baseline characteristics of RA and RA-ILD patients in the Cohort 1.

Variable	RA (*n* = 63)	RA-ILD (*n* = 46)	p-value
*Demographics and Clinical parameters*			
Age, years	56.95±10.18	58.09±12.36	0.612
Female gender, n (%)	47 (74.6%)	31 (67.4%)	0.542
Smoking history, n (%)	19 (30.2%)	16 (34.8%)	0.762
Tender joint count (TJC)	11.37±6.88	9.76±5.27	0.171
Swollen joint count (SJC)	2.06±1.32	2.00±1.17	0.792
Pain VAS, mm	33.50±14.20	39.80±15.80	0.032^a^
DAS28-ESR	5.06±0.89	5.20±0.79	0.382
*Hematological Parameters*			
White blood cells, ×10⁹/L	6.28 (5.21‒8.65)	7.76 (6.11‒10.41)	0.069
Red blood cells, ×10¹²/L	4.28±0.55	4.32±0.56	0.699
Hemoglobin, g/L	124.29±14.49	125.76±17.64	0.644
Platelets, ×10⁹/L	253.00 (218.00‒319.00)	235.50 (174.00‒283.00)	0.081
Neutrophils, ×10⁹/L	4.46 (3.33‒6.19)	5.20 (4.07‒7.13)	0.104
Lymphocytes, ×10⁹/L	1.39 (1.14‒1.78)	1.63 (1.12‒2.09)	0.077
Monocytes, ×10⁹/L	0.42 (0.32‒0.59)	0.50 (0.35‒0.63)	0.302
*Liver and Kidney Function Tests*			
ALT, U/L	17.30 (12.00‒24.60)	16.70 (12.90‒23.90)	0.874
AST, U/L	20.70 (17.60‒27.10)	24.50 (19.00‒28.70)	0.208
GGT, U/L	17.00 (14.20‒25.60)	22.85 (16.50‒32.40)	0.790
ALP, U/L	86.00 (65.80‒113.40)	95.00 (75.13–120.51)	0.017^a^
Total protein, g/L	71.83±5.37	69.69±7.72	0.110
Urea, LL	5.20 (4.19‒6.28)	5.63 (5.11‒7.21)	0.006^b^
Creatinine, μmoL/L	58.30 (49.90‒69.50)	69.25 (52.30‒78.20)	0.032^a^
Uric acid, μmoL/L	278.00 (214.00‒343.00)	309.50 (244.00‒364.00)	0.057
eGFR, mL/min/1.73m²	103.36(79.95‒126.17)	97.08(75.43‒122.25)	0.480
*Inflammatory and Serological Markers*			
hs-CRP, mg/L	6.60 (2.72‒17.50)	12.60 (4.53‒26.20)	0.508
ESR, mm/h	30.00 (16.00‒68.00)	42.00 (29.00‒70.00)	0.140
Rheumatoid factor, IU/mL	54.60 (20.00‒270.00)	212.00 (75.60‒475.00)	0.004^b^
Anti-CCP antibody, U/mL	253.00 (85.80‒413.00)	352.00 (100.40‒702.00)	0.018^a^
*Metabolic Parameters*			
Fasting glucose, mmoL/L	5.44 (4.98‒5.89)	5.18 (4.87‒5.84)	0.571
Total cholesterol, mmoL/L	5.04±1.82	5.06±1.04	0.948
Triglycerides, mmoL/L	1.22 (0.98‒1.58)	1.21 (0.87‒1.63)	0.794
HDL cholesterol, mmoL/L	1.43±0.33	1.53±0.47	0.232
LDL cholesterol, mmoL/L	2.99±0.76	3.12±0.63	0.337

Data are presented as mean ± SD, median (IQR), or n (%). RA, Rheumatoid Arthritis; RA-ILD, Rheumatoid Arthritis-Associated Interstitial Lung Disease; VAS, Visual Analog Scale; DAS28-ESR, Disease Activity Score in 28 joints using ESR; ALP, Alkaline Phosphatase; hs-CRP, high-sensitivity C-Reactive Protein; Anti-CCP, Anti-Cyclic Citrullinated Peptide. ^a^*p* < 0.05, ^b^*p* < 0.01, ^c^*p*<0.001.

The external validation cohort (Cohort 2) comprised 169 patients, including 102 with RA and 67 with RA-ILD (Table 2). The RA-ILD group exhibited significantly elevated tender joint counts, white blood cell counts, uric acid levels, total cholesterol, triglycerides, as well as CRP and anti-CCP levels, while HDL-C was notably reduced. No significant differences were observed in demographic characteristics or other clinical indicators between the two groups.

**Table 2:** Baseline characteristics of RA and RA-ILD patients in the cohort 2.

Variable	RA (*n* = 102)	RA-ILD (*n* = 67)	p-value
*Demographics and Clinical parameters*			
Age, years	58.24±10.85	59.87±11.26	0.327
Female gender, n (%)	24 (23.5%)	19 (28.4%)	0.486
Smoking history, n (%)	30 (29.4%)	21 (31.3%)	0.791
Tender joint count (TJC)	8.00 (7.00‒9.00)	10.00 (6.00‒14.00)	0.020^a^
Swollen joint count (SJC)	1.00 (0.00‒2.00)	1.00 (0.00‒2.00)	0.217
Pain VAS, mm	40.00 (20.00‒40.00)	30.00 (20.00‒40.00)	0.883
DAS28-ESR	5.05 (4.41‒5.38)	5.20 (4.61‒5.69)	0.086
*Hematological Parameters*			
White blood cells, ×10⁹/L	6.65 (5.59‒8.03)	7.69 (6.05‒8.99)	0.009^b^
Red blood cells, ×10¹²/L	4.17±0.52	4.19±0.52	0.805
Hemoglobin, g/L	118.82±16.21	118.79±17.05	0.990
Platelets, ×10⁹/L	252.50 (211.00‒309.00)	271.00 (227.00‒324.00)	0.372
Neutrophils, ×10⁹/L	4.54 (3.45‒5.81)	5.20 (4.07‒7.13)	0.052
Lymphocytes, ×10⁹/L	1.35 (1.13‒1.71)	1.43 (1.17‒2.01)	0.537
Monocytes, ×10⁹/L	0.41 (0.32‒0.58)	0.49 (0.39‒0.65)	0.087
*Liver and Kidney Function Tests*			
ALT, U/L	15.80 (11.40‒22.20)	14.40 (11.20‒19.90)	0.893
AST, U/L	22.50 (18.10‒26.50)	20.70 (16.50‒25.00)	0.109
GGT, U/L	19.75 (14.30‒28.70)	22.00 (15.60‒34.80)	0.935
ALP, U/L	86.00 (73.00‒106.00)	90.20 (67.00‒114.70)	0.095
Total protein, g/L	70.98±7.41	72.37±8.45	0.274
Urea, mmoL/L	5.68 (4.48‒6.83)	5.52 (4.78‒6.99)	0.720
Creatinine, μmoL/L	62.00 (50.60‒73.20)	63.30 (52.50‒72.10)	0.870
Uric acid, μmoL/L	259.50 (219.00‒339.00)	320.00 (248.00‒379.00)	0.002^b^
eGFR, mL/min/1.73m^2^	97.88 (83.34‒122.31)	100.65 (85.56‒115.60)	0.637
*Inflammatory and Serological Markers*			
hs-CRP, mg/L	10.14 (1.77‒31.40)	22.10 (10.00‒58.00)	0.015^a^
ESR, mm/h	46.00 (19.00‒81.00)	59.00 (35.00‒73.00)	0.262
Rheumatoid factor, IU/mL	55.05 (20.00‒313.00)	164.00 (20.00‒473.00)	0.193
Anti-CCP antibody, U/mL	187.50 (25.00‒419.00)	325.00 (28.00‒709.00)	0.021^a^
*Metabolic Parameters*			
Fasting glucose, mmoL/L	5.33 (4.91‒5.82)	5.34 (4.92‒5.93)	0.559
Total cholesterol, mmoL/L	4.86±1.09	5.55±1.20	<0.001^c^
Triglycerides, mmoL/L	1.24 (0.90‒1.52)	1.86 (1.57‒2.27)	<0.001^c^
HDL cholesterol, mmoL/L	1.45±0.42	1.22±0.36	<0.001^c^
LDL cholesterol, mmoL/L	2.88±0.79	2.98±0.93	0.454

Data are presented as mean ± SD, median (IQR), or n (%). RA, Rheumatoid Arthritis; RA-ILD, Rheumatoid Arthritis-Associated Interstitial Lung Disease; VAS, Visual Analog Scale; DAS28-ESR, Disease Activity Score in 28 joints using ESR; ALP, Alkaline Phosphatase; hs-CRP, high-sensitivity C-Reactive Protein; Anti-CCP, Anti-Cyclic Citrullinated Peptide. ^a^*p*<0.05, ^b^*p*<0.01, ^c^*p*<0.001.

### Multi-omics feature identification

The procedure for selecting radiomic features is depicted in Supplementary Figure S1. Regions of Interest (ROIs) were semi-automatically delineated under the lung window using 3D Slicer, and a substantial number of features were batch extracted using PyRadiomics, encompassing texture, morphological, and first-order statistical features. Ultimately, 1468 imaging features and their corresponding values were obtained. Following z-score normalization of the extracted feature values using the sapply function, dimensionality reduction was conducted through embedding based on a Vision Transformer self-supervised learning framework, culminating in the selection of the top 30 semantic features. Feature selection and optimization of radiomic features were executed using LASSO regression (Supplementary Fig. S2A‒B), resulting in the final identification of nine core imaging features for subsequent model construction.

Feature identification for transcriptomics, proteomics, and metabolomics was based on a significant differential omics feature analysis. After cross-selection of features using three machine learning algorithms ‒ SVM, RF, and LASSO (Supplementary Fig. S2C‒E) ‒ eight key genes, two core protein molecules, and five important metabolites were preliminarily identified.

### The CatBoost model for metabolomics achieved the best performance among single-omics models

**Fig. 2:**
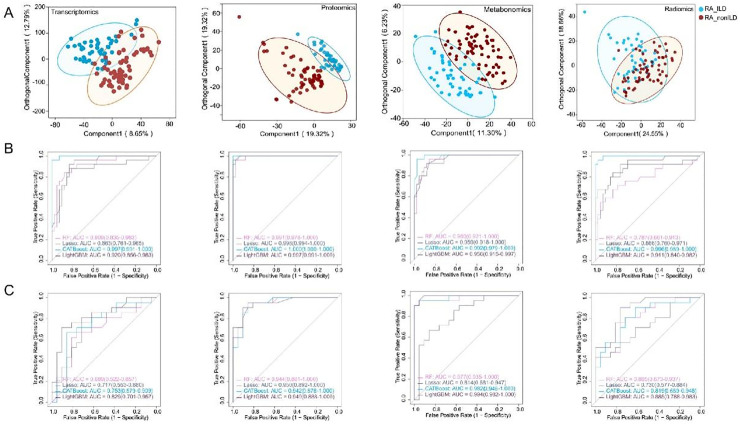
Single-omics analysis results: PLS-DA and machine learning model performance evaluation. (A) Partial Least Squares Discriminant Analysis (PLS-DA) score plots for radiomics, transcriptomics, proteomics, and metabolomics. (B) Receiver Operating Characteristic (ROC) curves of four machine learning algorithms (LASSO, RF, LightGBM, CatBoost) on the training sets of each omics dataset. (C) Corresponding ROC curves of the models on the validation set.

Partial Least Squares Discriminant Analysis (PLS-DA) results showed that among all omics data (Fig. 2A), radiomics had the best separation effect (Component 1 = 24.55%), transcriptomics had the lowest degree of separation (Component 1 = 8.65%), and proteomics and metabolomics were intermediate. All four omics displayed a trend of separation between RA and RA-ILD. Four machine learning algorithms (LASSO, RF, LightGBM, and CatBoost) were used to build diagnostic models, and their performances were evaluated using an independent validation set. The results showed that metabolomics performed the best overall (Fig. 2B‒C, Table 3), with the CatBoost model achieving an accuracy of 0.929, sensitivity of 0.857, and AUC of 0.982 (95% CI: 0.948–1.000). The proteomics models were consistently balanced and stable, with AUCs above 0.945 for all the algorithms. In radiomics models, LightGBM had the highest AUC (0.885), but its sensitivity was relatively low (0.571); the RF model had an accuracy of 0.738 and a Youden's index of 0.476. Overall classification performance of the transcriptomics models was limited, with the best AUC for LightGBM at 0.829, but sensitivities were mostly below 0.620, and the highest Youden's index for the CatBoost model was only 0.476. In conclusion, among the single-omics models, the metabolomics CatBoost model demonstrated the best performance.

**Table 3:** Performance evaluation of single-omics and multi-omics integration models.

	Models	Sensitivity	Specificity	Accuracy	PPV	NPV	F1	Youden's J	AUC_CI
**Radiomics**	RandomForest	0.714	0.762	0.738	0.750	0.727	0.732	0.476	0.805 (0.673‒0.937)
	Lasso	0.571	0.714	0.643	0.667	0.625	0.615	0.286	0.730 (0.577‒0.884)
	CATBoost	0.667	0.762	0.714	0.737	0.696	0.700	0.429	0.819 (0.689‒0.948)
	LightGBM	0.571	0.810	0.690	0.750	0.654	0.649	0.381	0.885 (0.788‒0.983)
**Metabolomics**	RandomForest	0.810	0.950	0.905	0.930	0.840	0.895	0.760	0.977 (0.935‒1.000)
	Lasso	0.762	0.667	0.714	0.696	0.737	0.727	0.429	0.814 (0.681‒0.947)
	CATBoost	0.857	0.960	0.929	0.940	0.875	0.923	0.817	0.982 (0.948‒1.000)
	LightGBM	0.619	0.950	0.810	0.920	0.724	0.765	0.569	0.994 (0.982‒1.000)
**Proteomics**	RandomForest	0.730	0.910	0.820	0.890	0.770	0.798	0.640	0.946 (0.885‒1.000)
	Lasso	0.700	0.895	0.795	0.870	0.745	0.772	0.595	0.950 (0.892‒1.000)
	CATBoost	0.740	0.920	0.830	0.900	0.780	0.812	0.660	0.945 (0.878‒1.000)
	LightGBM	0.710	0.900	0.805	0.880	0.755	0.783	0.610	0.948 (0.886‒1.000)
**Transcriptomics**	RandomForest	0.571	0.810	0.690	0.750	0.654	0.649	0.381	0.689 (0.522‒0.857)
	Lasso	0.524	0.762	0.643	0.688	0.615	0.595	0.286	0.717 (0.553‒0.880)
	CATBoost	0.619	0.857	0.738	0.812	0.692	0.703	0.476	0.753 (0.597‒0.909)
	LightGBM	0.429	0.952	0.690	0.900	0.625	0.581	0.381	0.829 (0.701‒0.957)
**Multi-omics Integration model**	RandomForest	0.810	0.950	0.950	0.905	0.840	0.895	0.760	0.984 (0.958‒1.000)
	Lasso	0.780	0.930	0.930	0.885	0.820	0.845	0.710	0.973 (0.936‒1.000)
	CATBoost	0.762	0.960	0.881	0.940	0.808	0.865	0.722	0.984 (0.959‒1.000)
	LightGBM	0.810	0.905	0.857	0.894	0.826	0.850	0.714	0.968 (0.927‒1.000)

### The performance of multi-omics integrated models surpasses that of single-omics models

**Fig. 3:**
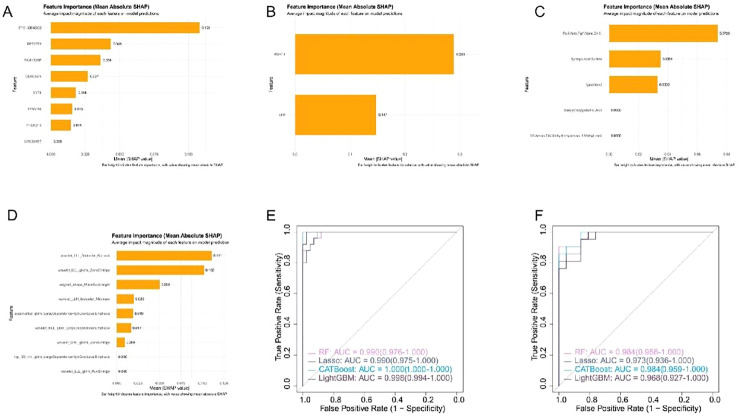
SHAP-based feature contribution explanations and multi-omics integration model performance. (A‒D) SHAP summary plots of the top predictive features in the optimal models of each single omics dataset. (E‒F) ROC curves of the multi-omics integration model, which incorporates TOP features from the four omics datasets above, on the (E) training set and (F) independent validation set.

To construct and optimize a diagnostic model integrating multiple omics data, the machine learning model with the highest AUC in the validation set was selected for each omics type. The SHAP interpretation framework was used to quantify the contribution of each feature to the prediction (Fig. 3A‒D). The most contributive features from the best-performing algorithms of each omics type were extracted to form a multi-omics feature set comprising four variables: radiomics wavelet_LLL_firstorder_Kurtosis, metabolomics 6-Keto-PGF1α, proteomics RDH11, and transcriptomics SYS1-DBNDD2. After integrating features from the four omics, the multi-omics model significantly surpassed the performance limitations of the single-omics models, exhibiting superior and more stable comprehensive discrimination capability than the optimal single-omics model in the validation set. All algorithms achieved an AUC of 0.968–0.984, with no low-performance models observed (Fig. 3E‒F, Table 3). In addition, the Positive Predictive Value (PPV) of the multi-omics model was consistently ≥0.885 and the Negative Predictive Value (NPV) was consistently ≥0.820, demonstrating a significant improvement in clinical reliability. These results indicate that a multi-omics strategy integrating molecular and imaging features outperforms single-omics approaches.

### Clinical-radiomics logistic regression model demonstrates good performance in both internal and external validation

**Fig. 4:**
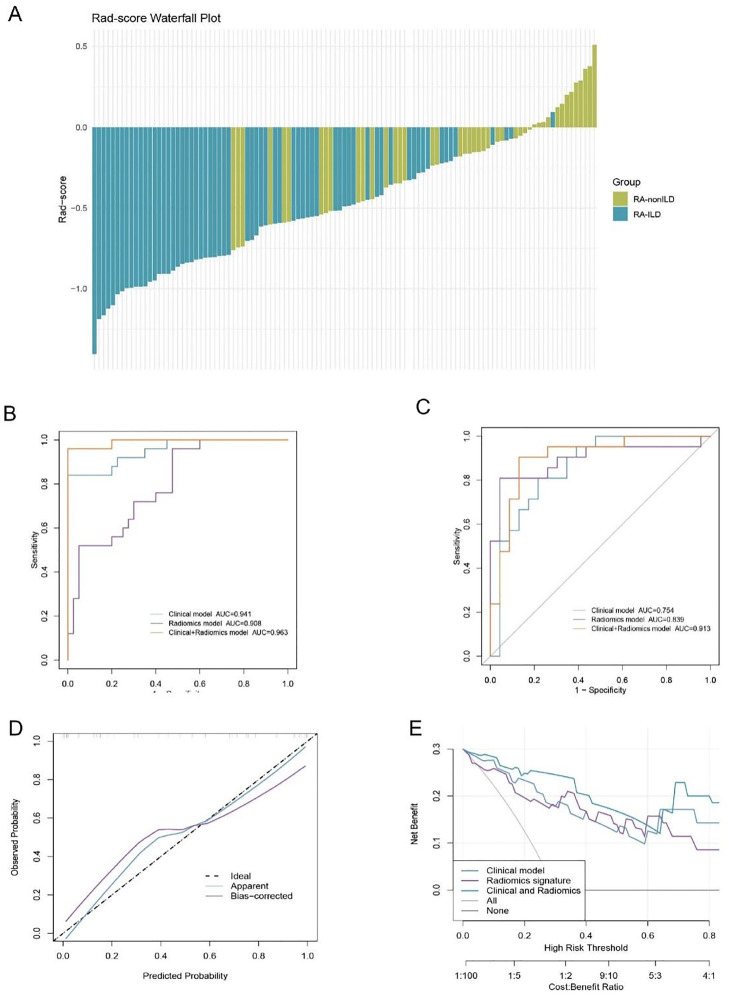
Waterfall plot of radiomics scores (Rad-score) and construction, validation, and clinical utility assessment of the radiomics-clinical logistic regression model. (A) Waterfall plot of the radiomics score (Rad-score) for each patient in the cohort. (B‒C) ROC curves of three models (clinical model, radiomics model, and combined clinical + radiomics model) in the (B) internal validation set and (C) multicenter external validation set. (D) Calibration curve of the clinical + radiomics model in the internal validation set. (E) Decision curve analysis (DCA) of the clinical + radiomics model.

To further enhance the clinical applicability and scalability of imaging models, the authors additionally developed a diagnostic model that integrates radiomics features with conventional clinical indicators. The Rad-score was calculated based on nine selected imaging features (Fig. 4A). The Rad-score for RA-ILD patients was significantly higher than that of RA-nonILD patients; the vast majority of RA-ILD cases were distributed in the high-value range, whereas RA-nonILD patients were concentrated in the low-value range. Subsequently, the authors extracted clinical indicators from Cohort 1 (Table 1) that showed intergroup differences (VAS score, ALP, urea, creatinine, RF, and anti-CCP) and combined these with the key radiomics Rad-score to construct an imaging-logistic regression model.

In internal validation, the Clinical-Radiomics fusion model exhibited the best diagnostic performance (Fig. 4A, Table 4), with an AUC of 0.963 and excellent sensitivity, specificity, and accuracy. In contrast, models using only radiomics or clinical features performed less effectively. During multi-center external validation, although all models showed some performance decline, the Clinical-Radiomics model maintained optimal stability: the AUC was 0.913, sensitivity reached 0.952, Youden's J index was 0.822, and both accuracy and F1 score were 0.886. Meanwhile, the performance of the Clinical model and Radiomics model declined to varying degrees in the external validation. These results confirm that the Clinical-Radiomics model can effectively resist the effects of sample heterogeneity across multiple centers and is significantly superior to single-feature models (Fig. 4B, Table 4). The calibration curve for the internal validation set (Fig. 4C) showed a high degree of agreement between the predicted probability and actual observed frequency, and decision curve analysis (Fig. 4D) further indicated that the model offers a substantial net clinical benefit across a wide range of threshold values. DCA results (Fig. 4E) further demonstrated that combining radiomics with clinical features can greatly enhance the differential diagnostic ability for RA-ILD.

**Table 4:** Performance evaluation of Clinical-Radiomics Logistic regression models in internal and external validation sets.

Validation Set	Model	Sensitivity	Specificity	Accuracy	AUC (95% CI)
Internal	Clinical model	0.840	0.850	0.870	0.941 (0.899‒1.000)
	Radiomics model	0.880	0.725	0.769	0.908 (0.688‒0.904)
	Clinical+Radiomics	0.960	0.980	0.972	0.963 (0.976‒1.000)
External	Clinical model	0.571	0.913	0.750	0.754 (0.619‒0.866)
	Radiomics model	0.810	0.957	0.886	0.839 (0.788‒1.000)
	Clinical+Radiomics	0.952	0.870	0.886	0.913 (0.808‒0.998)

**Fig. 5:**
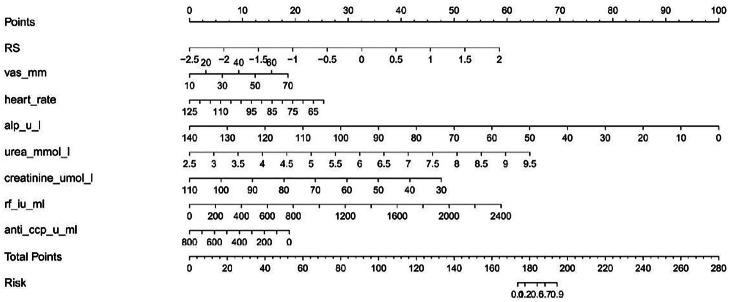
Nomogram for predicting RA-ILD risk based on multi-omics and clinical features.

To enable visual assessment of RA-ILD disease risk, the authors constructed a nomogram (Fig. 5) based on the optimal Clinical-Radiomics Logistic regression model. This nomogram includes two core types of variables: 1) The Rad-score, calculated from nine radiomics features selected by LASSO, and 2) Clinical indicators with intergroup differences (VAS score, ALP, urea, creatinine, RF, and anti-CCP). On the left side of the nomogram, the point range for each variable (Points) is indicated. The total score (Total Points, ranging from 0 to 260) is shown in the middle, and the corresponding RA-ILD risk (Risk, ranging from 0.1 to 0.9) is shown on the right. In clinical practice, users can look up and sum the corresponding scores based on patient test values and directly map the total score to a risk probability. For example, when the total score reaches 200, the RA-ILD risk approaches 0.9 (very high risk).

### Metabolite 6-Keto-PGF1αand genes SYS1-DBNDD2 are closely related to inflammatory activation and autoimmune responses

Intergroup comparisons (Supplementary Fig. S3A‒D) showed significant differences in the four key features between the RA-ILD and RA groups. Spearman correlation analysis (Supplementary Fig. S4, Supplementary Fig. S5A‒F) revealed that the metabolite 6-Keto-PGF1αexhibited a significant positive correlation with various inflammatory markers, including CRP, neutrophil count, anti-CCP antibody levels, and Neutrophil-to-Lymphocyte Ratio (NLR). The expression level of the transcript SYS1-DBNDD2 also showed a significant positive correlation with neutrophil count and Systemic Immune-Inflammation Index (SII). No significant associations (after FDR correction) were found between the radiomic feature Kurtosis or the protein RDH11 and the inflammatory indicators analyzed.

### Key multi-omics features are associated with pulmonary function imbalance in RA-ILD

Spearman correlation analysis results showed (Supplementary Fig. S6, Supplementary Fig. S7A‒F) that the key radiomics feature Kurtosis was significantly negatively correlated with DLCO%pred, which represents gas exchange efficiency (Supplementary Fig. S7A), indicating that the higher the value of this imaging feature, the worse the diffusion function in patients. Meanwhile, Kurtosis showed a significant positive correlation with the Warrick score, which reflects the degree of pulmonary fibrosis (Supplementary Fig. S7B), as well as with the CPI index, which comprehensively evaluates physiological impairment (Supplementary Fig. S7C), suggesting that this feature is associated with more severe radiological and physiological lesions. The concentration of the proteomic biomarker RDH11 was significantly negatively correlated with the DLCO/FVC ratio (Supplementary Fig. S7D), indicating that lower RDH11 levels may, to some extent, suggest impaired gas exchange function after lung volume correction. Although the metabolite 6-Keto-PGF1αand transcript SYS1-DBNDD2 showed a certain trend of association with some indicators, neither reached statistical significance after FDR correction.

### Biological functions involving key imaging features

**Fig. 6:**
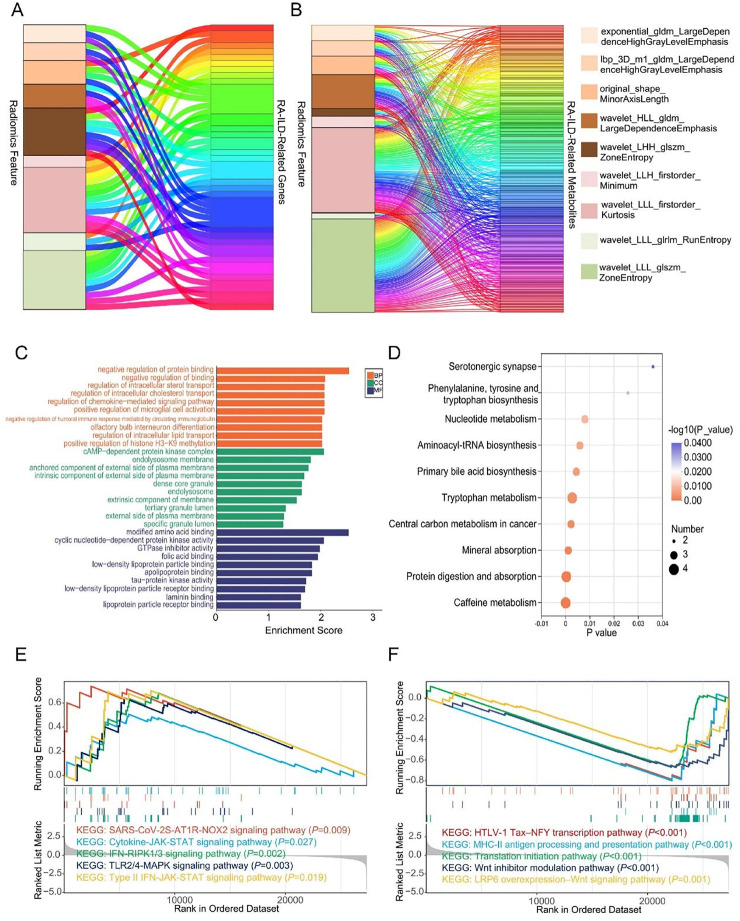
Multi-omics correlation and functional enrichment analysis linking radiomic features to biological functions. (A‒B) Sankey diagrams illustrate the correlation networks between 9 key radiomic features and significantly altered genes (left) and metabolites (right). (C) GO enrichment of differentially expressed genes correlated with imaging features. (D) KEGG pathway enrichment of differentially expressed metabolites correlated with imaging features. (E‒F) Gene Set Enrichment Analysis (GSEA) based on groups divided by the median value of wavelet_LLL_firstorder_Kurtosis.

Next, the authors conducted an in-depth investigation into the biological functions of the imaging model features. The Sankey diagrams (Fig. 6A‒B) systematically present the correlation network among gene features, metabolite features, and the nine core radiomics features of RA-ILD. Spearman correlation analysis showed that the nine key imaging model features have significant associations with 48 genes and 356 metabolites (*p* < 0.05). Among these, the features wavelet_LLL_firstorder_Kurtosis and wavelet_LLL_glszm_ZoneEntropy occupy central positions and exhibit significant differences between the two groups. These key imaging features are also connected to multiple genes involved in the regulation of intracellular lipid transport and metabolites in the tryptophan metabolic pathway.

GO enrichment analysis of differentially expressed genes associated with key imaging features (Fig. 6C) indicated that these genes were significantly enriched in 161 pathways at the level of Biological Processes (BP), mainly involving immune regulation, lipid and steroid transport regulation, and signaling pathway modulation; in 8 pathways at the Cellular Component (CC) level, focusing on cell structure and organelle components; and in 25 pathways at the Molecular Function (MF) level, covering molecular binding and enzyme activity regulation. KEGG enrichment analysis of differentially expressed metabolites linked to key imaging features revealed (Fig. 6D) that tryptophan metabolism and primary bile acid biosynthesis were the two most significantly enriched core pathways. Additionally, a range of amino acid metabolic pathways, including phenylalanine, tyrosine, and tryptophan biosynthesis, aminoacyl-tRNA biosynthesis, and protein digestion and absorption, were significantly associated with imaging abnormalities.

The GSEA results (Fig. 6E) showed that immune-inflammatory and fibrosis-related pathways, such as the TLR signaling pathway, JAK-STAT signaling pathway, and cytokine receptor interactions, were closely associated with high Kurtosis imaging features. In contrast, tissue repair-and immune regulation-related pathways, such as the “Wnt signaling pathway” and “antigen processing and presentation”, are associated with low Kurtosis imaging features.

## Discussion

The early diagnosis of RA-ILD continues to face challenges due to the lack of sensitive and specific biomarkers and the subjectivity in imaging interpretation. Based on this, the present study first screened key features from omics data to construct single-omics machine learning diagnostic models and identified the optimal omics model. Subsequently, by integrating core features from multiple omics, the authors built a multi-omics integration model that significantly enhanced the early identification of RA-ILD. In addition, an imaging-clinical logistic regression model was established using radiomic features and clinical indicators. Validated with multicenter data, this model demonstrated outstanding diagnostic performance and good generalizability in the present study. Finally, the authors explored the close association between the selected key multi-omics features and systemic or specific pathway disruption. These multi-omics biomarkers showed multidimensional associations with lung function and inflammation, providing new evidence for establishing a complete pathological pathway from molecular abnormalities to physiological dysfunction in RA-ILD.

Notably, among the single-omics approaches evaluated in this study, the metabolomics model demonstrated the most superior diagnostic performance. This finding aligns with the biological hierarchy, where the metabolome serves as the ultimate downstream phenotypic readout of the central dogma, reflecting the cumulative effects of genetic regulation, enzymatic activities, and environmental interactions.^16^ In complex heterogeneous diseases like RA-ILD, metabolic signatures may capture subtle, real-time physiological perturbations more acutely than upstream transcriptomic or proteomic changes, which can be subject to regulatory buffering or post-translational modifications.^17^ The present results specifically identified significant enrichments in tryptophan metabolism and lipid-related pathways, which are consistent with recent advancements in pulmonary fibrosis research.^18^^,^^19^ Recent studies have highlighted that metabolic reprogramming is a hallmark of fibrotic lung diseases, where shifts in amino acid metabolism, particularly the tryptophan-kynurenine pathway, play a critical role in modulating immune tolerance and fibroblast activation.^20^ Furthermore, the prominence of lipid mediators in this model supports emerging evidence that bioactive lipids are essential signaling molecules driving the transition from inflammation to fibrosis in autoimmune conditions.^21^ By capturing these distinct metabolic alterations, the metabolomics model offers a robust reflection of the active disease state, explaining its higher sensitivity and accuracy compared to other single-omics modalities.

The multi-omics integration model and clinical-imaging nomogram constructed in this study exhibited excellent performance in diagnosing RA-ILD. Compared to existing studies, the strength of the studied model lies in its integration of multi-omics and imaging data, offering a more comprehensive view of the disease than models based on a single data source. For instance, in molecular subtyping studies of breast cancer similar to ours, the MOFA + model, which integrates transcriptomic, epigenomic, and microbiome data, achieved an F1 score of 0.75 and identified 121 key pathways, markedly outperforming the single-omics models.^22^ The multi-omics integration strategy employed in this study also significantly improved diagnostic robustness, reaffirming the complementary value of multi-omics data in capturing disease heterogeneity. Furthermore, compared to other multi-omics integration methods, the SHAP value-based feature selection strategy integrates the TOP1 feature from each model, thus ensuring performance while enhancing clinical interpretability. Although deep learning tools, such as Flexynesis, can integrate multi-omics and imaging data, their “black box” nature restricts their clinical trust and application. Likewise, the large radiotherapy model from Shandong First Medical University, while achieving multimodal fusion, focuses on prognosis prediction rather than diagnosis. In contrast, the present study quantifies feature contributions through SHAP values, transforming complex models into interpretable biological markers, a strategy similar to MOFA+'s clinical variable correlation with latent factors, and is more conducive to rapid clinical translation.^23^

Among the selected biomarkers, the significant correlation between the radiomics feature wavelet_LLL_firstorder_Kurtosis and pulmonary function indicators (DLCO%) as well as imaging severity scores (Warrick score, CPI index), indicates that this feature not only distinguishes disease states but also quantifies the structural changes in the lung parenchyma and their functional consequences. This is consistent with previous studies confirming that radiomic features can predict declines in lung function.^24^^,^^25^ Moreover, Kurtosis not only shows significant differences between groups but also occupies a central position in Sankey diagram analysis and pathway enrichment: the high Kurtosis group is enriched in pro-inflammatory and pro-fibrotic pathways such as TLR-MAPK and cytokine-JAK-STAT,^26^^,^^27^ while the low Kurtosis group demonstrates suppression of tissue repair pathways (Wnt signaling).^28^ This suggests that this feature reflects the underlying molecular imbalance in RA-ILD and provides strong evidence for the biological interpretation of radiomic biomarkers. From a clinical perspective, given its ability to quantify tissue heterogeneity beyond gross anatomical changes, Kurtosis could serve as an objective surrogate endpoint for monitoring responses to antifibrotic therapies (e.g., nintedanib).^29^ Unlike pulmonary function tests, which may show delayed responsiveness, tracking radiomic trajectory might allow clinicians to detect structural stabilization earlier in the treatment course.

High expression of the transcriptome marker SYS1-DBNDD2 and its positive correlation with neutrophil count and the SII index provide new clues for understanding the immune mechanisms of RA-ILD. The SYS1 gene is involved in regulating intracellular vesicle transport and may affect the secretion of inflammatory factors^30^; DBNDD2 has been suggested in some studies to be associated with the activation of the mTOR signaling pathway, which is critical for fibroblast activation and immune cell function.^31^^,^^32^ The co-high expression of both may synergistically promote antibody production and neutrophil-mediated inflammatory responses, which aligns with the enhanced autoimmunity and chronic inflammation observed in RA-ILD patients. Previous studies have mostly focused on the roles of cytokines such as IL-6 and TNF-α in RA-ILD,^14^^,^^33^ while these findings suggest that endogenous gene expression regulation may be another key aspect driving the inflammatory phenotype. Specifically, persistent elevation of SYS1-DBNDD2 despite conventional treatment could signal the need for agents targeting alternative pathways, such as JAK inhibitors or biological DMARDs, to dampen the neutrophil-driven inflammatory axis.^34^

The proteomic marker RDH11 is a key enzyme involved in retinol metabolism. Its downregulation may lead to insufficient retinoic acid synthesis, thereby weakening the inhibition of fibroblast activation.^35^^,^^36^ The negative correlation between RDH11 and the DLCO/FVC ratio further supports the potential role of abnormal retinol metabolism in the progression of pulmonary fibrosis,^37^^,^^38^ which is consistent with our finding that RDH11 expression is negatively correlated with the degree of fibrosis. Clinically, monitoring the restoration of RDH11 levels could offer a specific molecular readout for the efficacy of treatments aimed at restoring epithelial integrity, distinguishing true tissue repair from mere symptomatic relief.

The metabolite 6-Keto-PGF1α was found to be significantly elevated in RA-ILD patients, and shows a significant positive correlation with multiple inflammatory markers, including CRP, neutrophil count, anti-CCP antibody levels, and Neutrophil-Lymphocyte Ratio (NLR). 6-Keto-PGF1α is a stable metabolite of prostacyclin (prostaglandin I2, PGI2), which is known as a potent vasodilator and inhibitor of platelet aggregation, and also plays a role in the regulation of inflammation. Previous studies have shown that the prostaglandin pathway is abnormally activated in fibrotic diseases. In patients with Idiopathic Pulmonary Fibrosis (IPF),^39^ the expression of prostaglandin synthase in lung tissue is elevated, and animal models have confirmed its involvement in the regulation of fibroblast proliferation and collagen deposition. Crucially, 6-Keto-PGF1α is a downstream product of the Cyclooxygenase (COX) pathway, which is the primary target of NSAIDs, and corticosteroids commonly prescribed to RA patients. The paradoxical elevation of this metabolite observed in the studied cohort, despite the widespread use of these anti-inflammatory drugs, suggests that the pathological drive in RA-ILD overrides standard pharmacological suppression. Thus, 6-Keto-PGF1α may serve as a sensitive marker of “residual inflammatory risk”, where failure to normalize levels indicates a need for therapeutic escalation. The present study is the first to link 6-Keto-PGF1α with inflammatory activity in RA-ILD, suggesting that it may serve as a key molecule connecting vascular abnormalities, inflammatory responses, and pulmonary fibrosis. Elevated 6-Keto-PGF1α may reflect a compensatory mechanism by the body to counteract platelet activation or vasoconstriction; however, its coexistence with a pro-inflammatory state reveals the complex imbalance between inflammation and repair pathways in RA-ILD.^14^

However, this study is subject to several limitations that should be addressed systematically. First, the potential confounding influence of pharmacological treatments represents a significant challenge. Since RA patients are typically managed with complex and varying medication regimens ‒ including corticosteroids, conventional synthetic DMARDs, and biological agents ‒ it is difficult to completely disentangle the metabolic and proteomic alterations driven specifically by ILD pathology from those induced by long-term drug exposure. Although the authors attempted to match baseline characteristics, the heterogeneity in medication history could still influence the expression levels of the identified biomarkers, particularly the metabolic profiles. Future studies should aim to stratify patients based on treatment subgroups to rule out drug-induced metabolic artifacts. Second, regarding sample size, although the authors utilized an external cohort for validation, the total number of included cases, particularly for the high-dimensional omics analysis, remains relatively small. This limitation may constrain the statistical power to detect subtler molecular signals and could introduce potential selection bias. The current sample size may not fully capture the complete heterogeneity of the RA-ILD population, and therefore, these findings require further verification in larger, prospective, multi-center populations to ensure their generalizability and robustness. Finally, the cross-sectional design of this study inherently limits the ability to infer causality. While the authors identified strong associations between multi-omics features and disease status, longitudinal tracking is necessary to determine whether these key biomarkers are drivers of disease progression or merely downstream consequences of pulmonary injury.

## Conclusion

This study integrates machine learning, multi-omics, and radiomics data to construct a high-performance diagnostic model that significantly enhances the early identification of RA-ILD. The identified multi-omics potential biomarkers are expected to reveal the pathophysiological processes of RA-ILD from multiple dimensions, laying a new foundation for early screening and targeted therapy research.

## Data availability

All data generated during this study are included in the published article and its Supplementary information files.

## Ethics statement

This study was conducted in accordance with the Declaration of Helsinki and approved by the Ethics Committee of the First Affiliated Hospital of Army Medical University [(B) KY2025065)]. All participating centers obtained local institutional review board approval before patient recruitment. Written informed consent was obtained from all participants prior to enrollment, including consent for the collection and analysis of clinical data, medical imaging, and biological samples for research purposes.

All patient data were de-identified and anonymized to protect patient privacy and confidentiality. The study protocol was reviewed and approved by the ethics committee. The collection and processing of blood samples for transcriptomic, proteomic, and metabolomic analyses were performed in accordance with institutional guidelines for human biospecimen research. All clinical data were stored securely with access restricted to authorized research personnel only. The study complied with all applicable local and national regulations regarding clinical research involving human subjects. This study was registered in the Chinese Clinical Trial Registry (PID: 269,767, ChiCTR2500102339, Reg Date: 2025–05–13). The study design and reporting comply with the STARD guidelines for diagnostic accuracy studies (registration number: ChiCTR2500102339).

## Consent for publication

Not required.

## Authors’ contributions

Dandan Wu: Writing-original draft; Visualization; Software; Methodology; Investigation; Formal analysis; Data curation. Jianghua Chen: Investigation; Formal analysis; Conceptualizations. Heng Liang: Investigation; Data curation; Supervision. Cong Chen, Mei Liang, Cuiting Liao, Xueke He: Data curation; Supervision. Jiansheng, Zhai, Min Dai, Xiaorong Lu: Supervision. Fanxin Zeng: Writing-review & editing; Validation; Supervision; Conceptualization. Qinghua Zou: Writing-review & editing; Validation; Supervision; Conceptualization.

## Funding

This study was supported by the Chongqing Municipal Health Commission's “Key Flagship Department of Integrated Traditional Chinese and Western Medicine”: the 2023 Central Government Subsidy Fund for Traditional Chinese Medicine in Chongqing (525Z286) and Traditional Chinese Medicine Research Program of the Chongqing Municipal Health Commission, China (2025WSJK123).

## Declaration of competing interest

The authors declare that they have no known competing financial interests or personal relationships that could have appeared to influence the work reported in this paper.

## Appendix Supplementary materials

Image, application 1

Image, application 2

Image, application 3

Image, application 4

Image, application 5

Image, application 6

Image, application 7
