# Supplementary material for: Construction of Rheumatoid Arthritis-Associated Interstitial Lung Disease diagnostic model and identification of biomarkers based on a multi-omics integration strategy of machine learning
Source: Clinics (Sao Paulo). 2026 Apr 17;81:100933. doi: 10.1016/j.clinsp.2026.100933 (PMC13098433; doi:10.1016/j.clinsp.2026.100933)

**CLINICS-D-25-01940**

**Supplementary Materials**

**Supplementary Figure S1** Workflow of radiomics feature extraction from HRCT images. Steps include image preprocessing, ROI segmentation, and extraction of texture, first-order, filter-based, and shape features. A total of 1,468 features were initially extracted, reduced to 9 key features via LASSO and Transformer. Created with BioGDP.com.


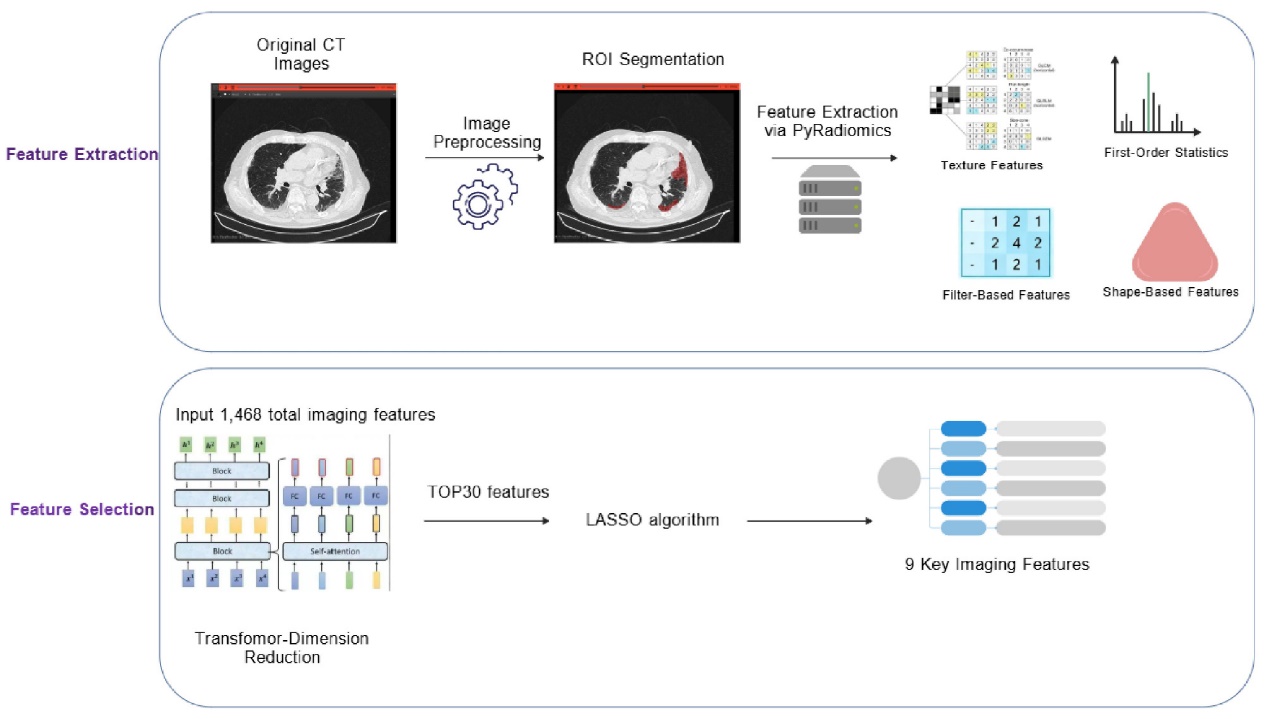


**Supplementary Figure S2** Screening of candidate diagnostic biomarkers and differential radiomic features based on multi-algorithm consensus. (A) Selection process of the tuning parameter (λ) for radiomic feature selection in the LASSO regression model. (B) LASSO coefficient path diagram of the radiomic features. The vertical line at the optimal λ value identifies nine features with non-zero coefficients. (C‒E) Venn diagrams illustrating the consensus features identified by multiple algorithms (LASSO, SVM, RF) for the (C) transcriptomics, (D) proteomics, and (E) metabolomics datasets, respectively.


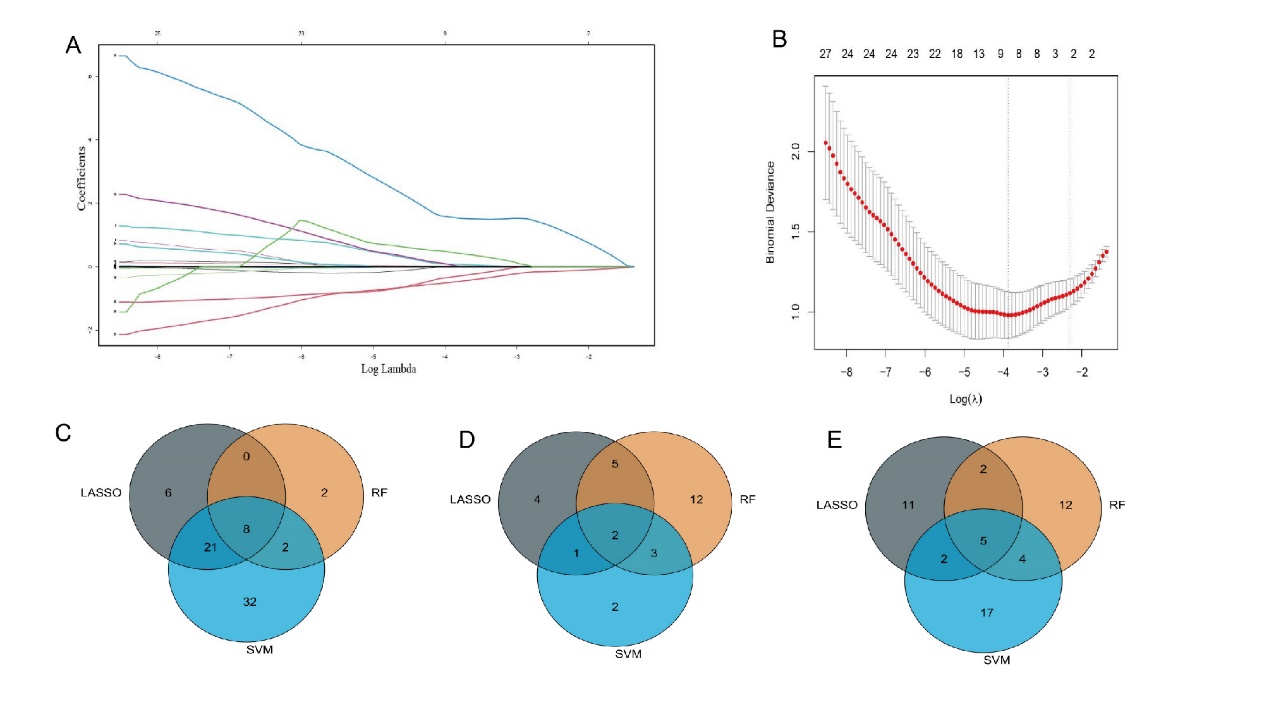


**Supplementary Figure S3** Distribution of top multi-omics features between RA and RA-ILD groups. Box plots show significant differences in radiomics kurtosis, RDH11, 6-Keto-PGF1a, and SYS1-DBNDD2 with Cohen's *d* effect size and p-values. (A) Radiomics feature: Kurtosis. (B) Proteomics feature: RDH11. (C) Metabolomics feature: 6-Keto-PGF1α. (D) Transcriptomics feature: SYS1-DBNDD2. Data are shown as mean ± SD (* p < 0.05, ** p < 0.01, *** p < 0.001).


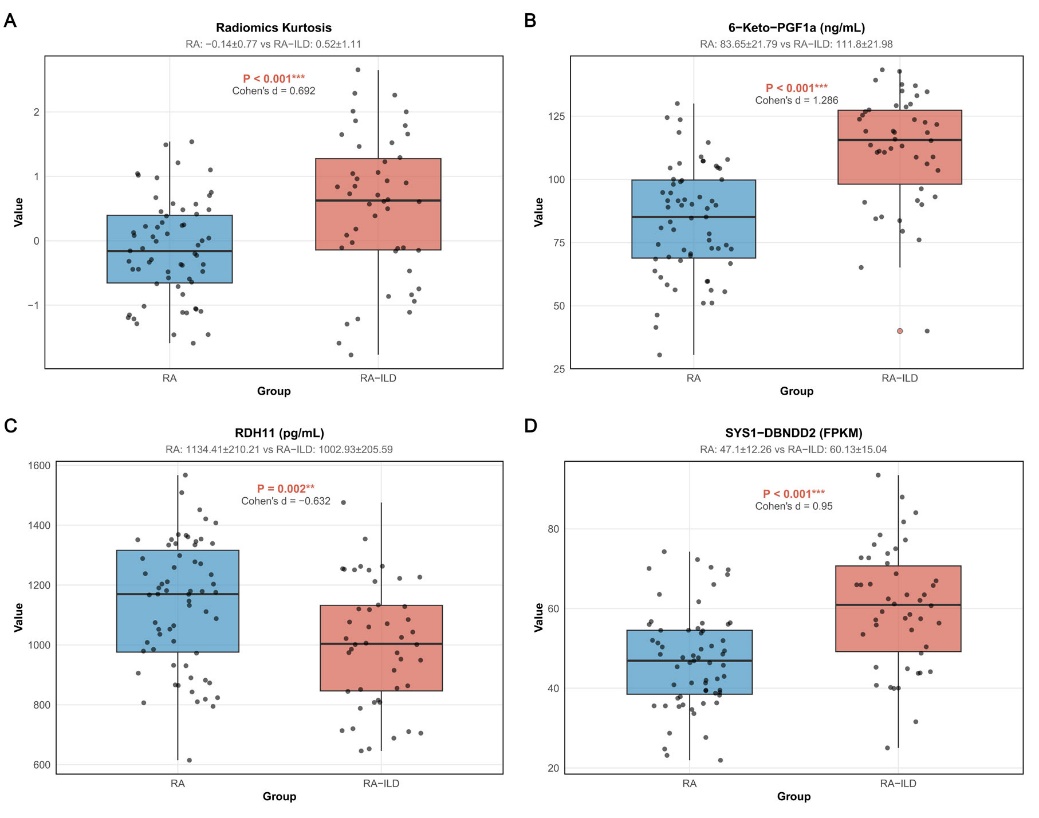


**Supplementary Figure S4** Spearman correlation heatmap between the top multi-omics features and systemic inflammatory indicators across the entire cohort. The color scale represents the strength and direction of the Spearman's correlation coefficient (ρ), where red indicates a positive correlation and blue indicates a negative correlation. The intensity of the color corresponds to the magnitude of the correlation. Significance levels after False Discovery Rate (FDR) correction are denoted as follows: * p < 0.05, ** p < 0.01, *** p < 0.001.

**
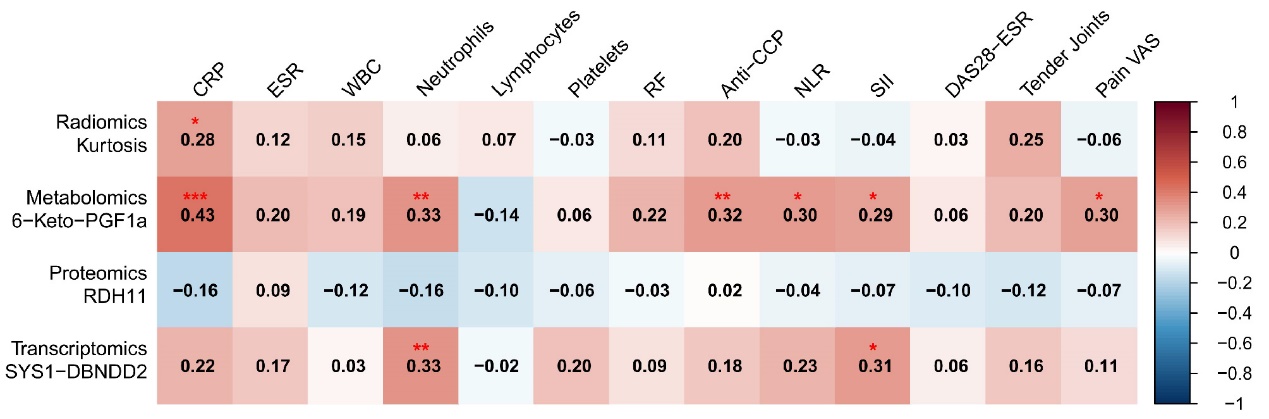
**

**Supplementary Figure S5** Scatter plots showing key correlations between multi-omics features and inflammatory markers in the overall cohort. Spearman's correlation coefficient (ρ) and False Discovery Rate (FDR) ‒ corrected p-values are indicated for each plot. Each point represents an individual patient, color-coded by diagnosis (blue: RA; red: RA-ILD). (A) 6-Keto-PGF1α vs. C-Reactive Protein (CRP). (B) SYS1-DBNDD2 gene expression vs. Neutrophil count. (C) 6-Keto-PGF1α vs. Neutrophil count. (D) 6-Keto-PGF1α vs. Anti-Cyclic Citrullinated Peptide (Anti-CCP) antibody level. (E) SYS1-DBNDD2 gene expression vs. Systemic Immune-inflammation Index (SII). (F) 6-Keto-PGF1α vs. Neutrophil-to-Lymphocyte Ratio (NLR).


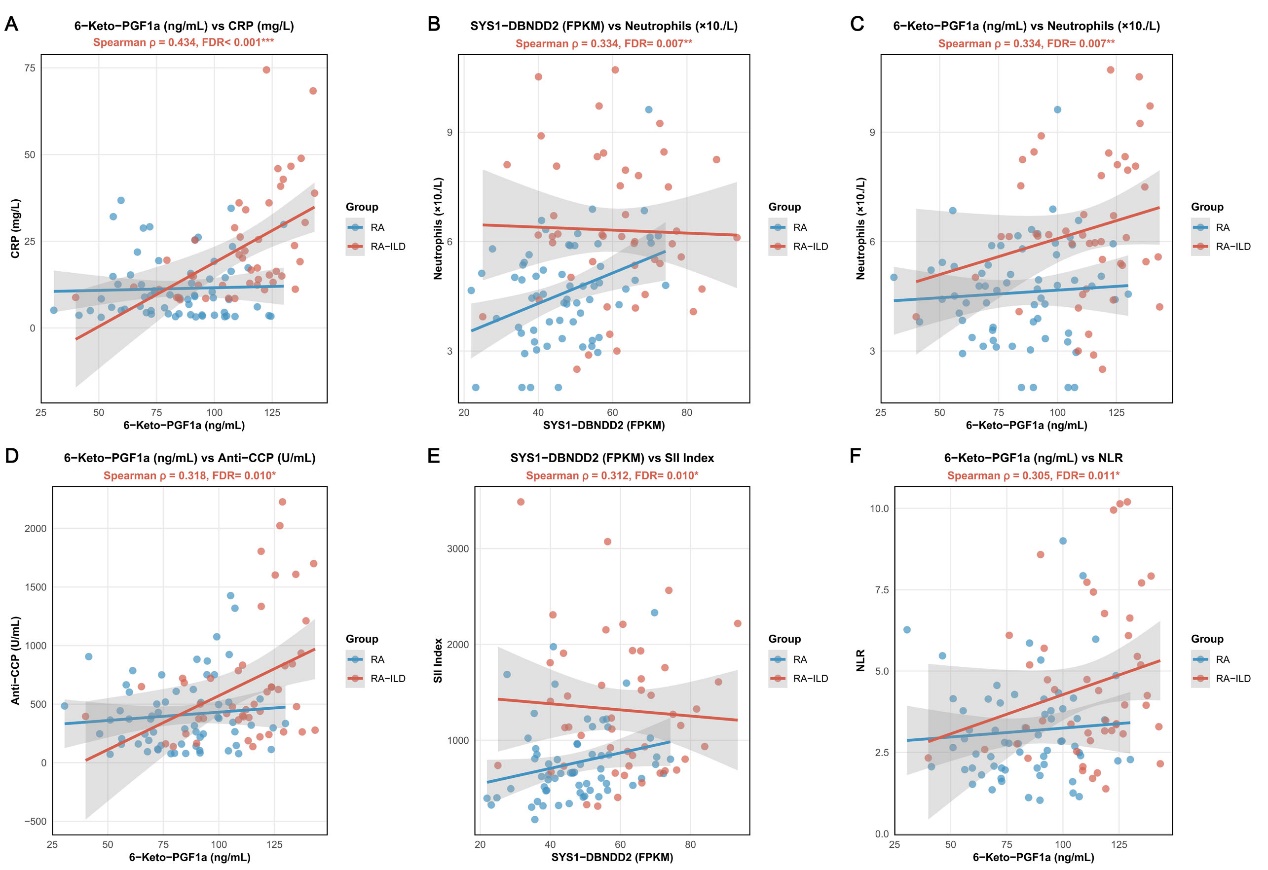


**Supplementary Figure S6** Spearman correlation heatmap between the top multi-omics features and pulmonary function/imaging indices in the RA-ILD cohort. The color scale represents the strength and direction of the Spearman's correlation coefficient (ρ), where red indicates a positive correlation and blue indicates a negative correlation. Significance levels after False Discovery Rate (FDR) correction are denoted as follows: * p < 0.05, **p < 0.01, *** p < 0.001.


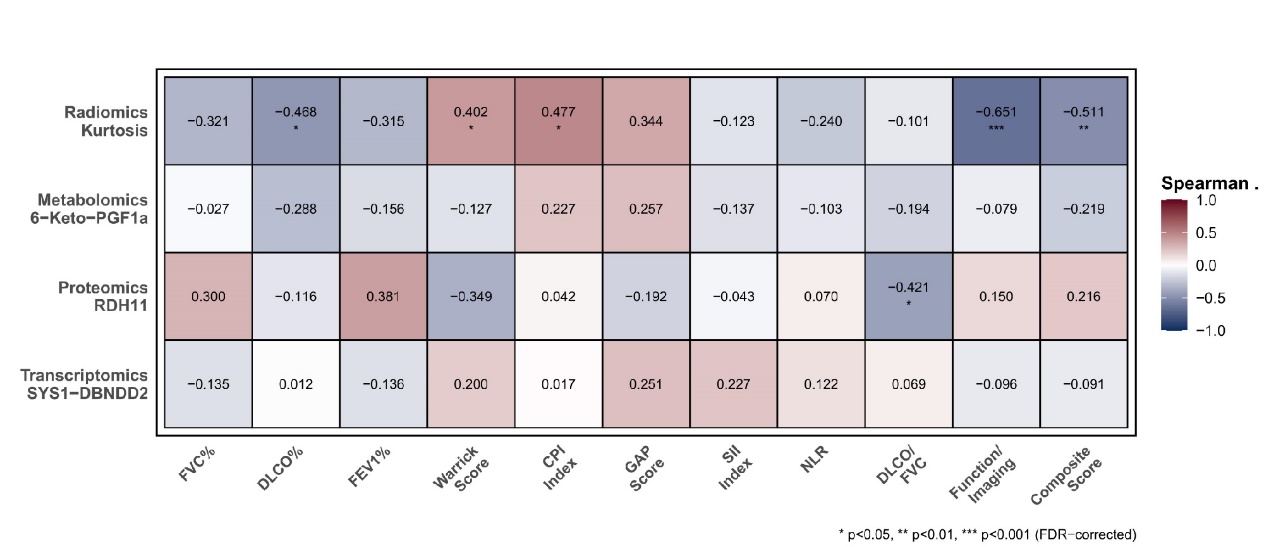


**Supplementary Figure S7** Key correlations between multi-omics features and pulmonary function/imaging indices in the RA-ILD cohort. Scatter plots with regression lines illustrate significant associations. Spearman's correlation coefficient (ρ) and FDR-corrected p-values are indicated for each plot. Each point represents an individual RA-ILD patient. (A) Radiomics Kurtosis vs. DLCO (% predicted). (B) Radiomics Kurtosis vs. Warrick Score. (C) Radiomics Kurtosis vs. CPI (Composite Physiologic Index). (D) RDH11 (Proteomics) vs. DLCO/FVC Ratio. (E) Radiomics Kurtosis vs. Function/Imaging Ratio (DLCO %pred/Warrick Score). (F) Radiomics Kurtosis vs. Composite Pulmonary Score.


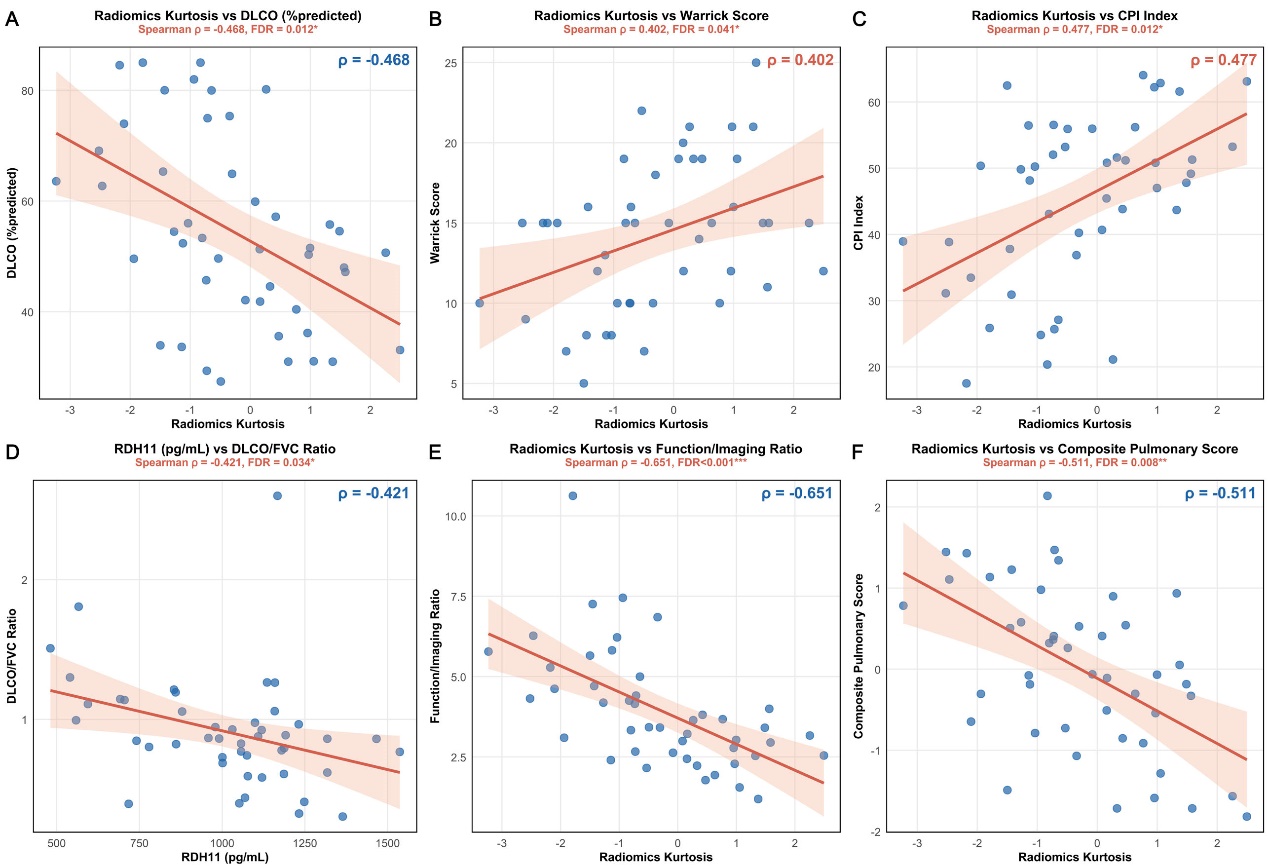

Supplement: Supplementary file 1 [file mmc1.docx]
