# Supplementary material for: Construction of Rheumatoid Arthritis-Associated Interstitial Lung Disease diagnostic model and identification of biomarkers based on a multi-omics integration strategy of machine learning
Source: Clinics (Sao Paulo). 2026 Apr 17;81:100933. doi: 10.1016/j.clinsp.2026.100933 (PMC13098433; doi:10.1016/j.clinsp.2026.100933)
Supplement: Supplementary file 2 [file mmc2.docx]

**Supplementary Table 1**

**Comprehensive listing of candidate image features, genes,proteins and metabolites identified in the study.**

#### Image Features

| **Feature Name** | **Category** |
| --- | --- |
| original_shape_MinorAxisLength | Shape feature |
| exponential_gldm_LargeDependenceHighGrayLevelEmphasis | Texture feature |
| lbp_3D_m1_gldm_LargeDependenceHighGrayLevelEmphasis | Texture feature |
| wavelet_LHH_glszm_ZoneEntropy | Texture feature |
| wavelet_HLL_gldm_LargeDependenceEmphasis | Texture feature |
| wavelet_LLH_firstorder_Minimum | First-order statistics |
| wavelet_LLL_firstorder_Kurtosis | First-order statistics |
| wavelet_LLL_glrlm_RunEntropy | Texture feature |
| wavelet_LLL_glszm_ZoneEntropy | Texture feature |

#### Genes

| **Gene ID** | **Gene Name** |
| --- | --- |
| ENSG00000254806 | SYS1-DBNDD2 |
| ENSG00000258116 | PPIAP45 |
| ENSG00000227081 | RPS27P3 |
| ENSG00000249140 | PRDX2P3 |
| ENSG00000230562 | FAM133DP |
| ENSG00000149043 | SYT8 |
| ENSG00000197503 | LINC00477 |
| ENSG00000105388 | CEACAM5 |

#### Proteins

| **Protein Name** | **Function** |
| --- | --- |
| RDH11 | Retinol dehydrogenase 11 |
| LPP | Lipoma-preferred partner |

#### Metabolites

| **Metabolite Name** | **Type** |
| --- | --- |
| Pc(6 Keto-Pgf1Alpha/24:0) | Phosphatidylcholine |
| 6-Amino-5[N-Methylformylamino]-1-Methyluracil | Purine metabolite |
| Deoxycholic Acid | Bile acid |
| Iguratimod | Synthetic compound |
| Syringic Acid Sulfate | Phenolic compound |
