# Supplementary material for: Construction of Rheumatoid Arthritis-Associated Interstitial Lung Disease diagnostic model and identification of biomarkers based on a multi-omics integration strategy of machine learning
Source: Clinics (Sao Paulo). 2026 Apr 17;81:100933. doi: 10.1016/j.clinsp.2026.100933 (PMC13098433; doi:10.1016/j.clinsp.2026.100933)
Supplement: Supplementary file 4 [file mmc4.docx]

**Supplementary Figure 1: Box plot illustrating the distribution of nine radiomics features selected by LASSO in the RA-ILD and RA-only groups.** (A) Four features show significant differences between groups. (B) Five features do not show significant differences between groups.


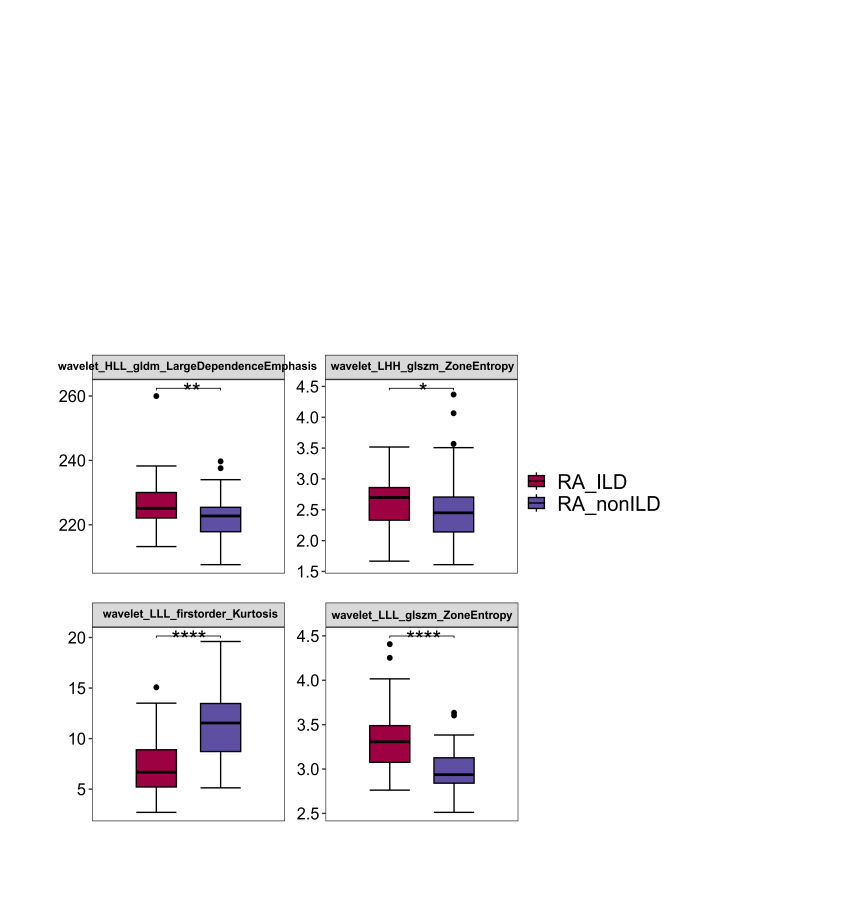

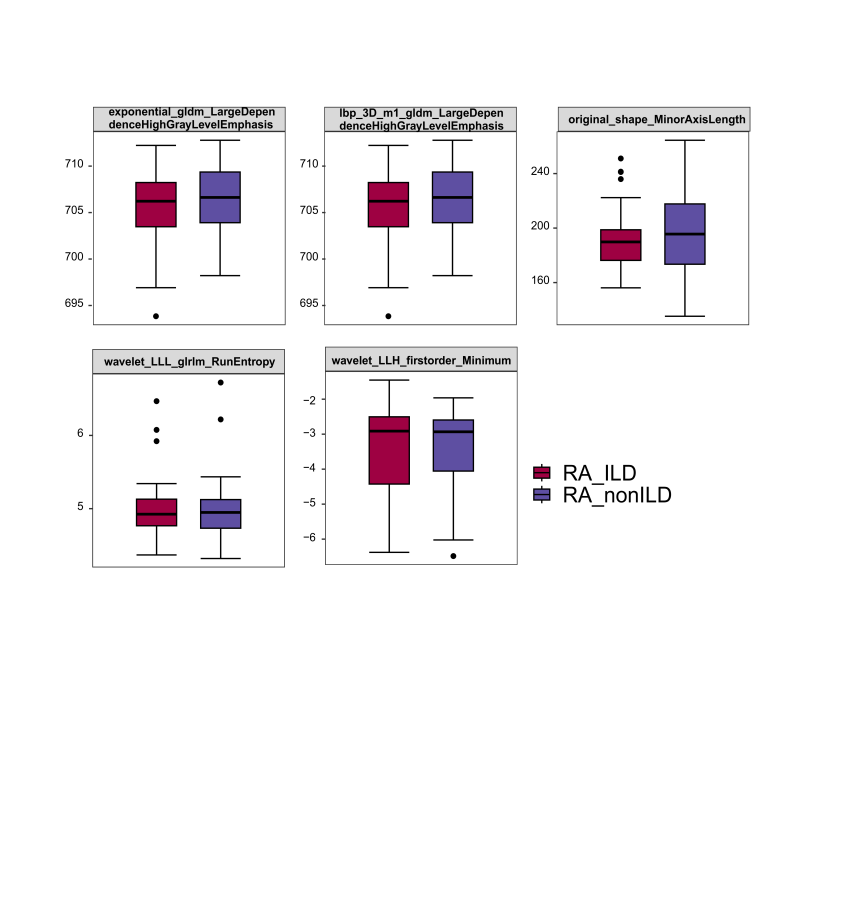


A

B
